# Complete genome sequence of *Tolumonas auensis* type strain (TA 4^T^)

**DOI:** 10.4056/sigs.2184986

**Published:** 2011-09-23

**Authors:** Olga Chertkov, Alex Copeland, Susan Lucas, Alla Lapidus, Kerrie W. Berry, John C. Detter, Tijana Glavina Del Rio, Nancy Hammon, Eileen Dalin, Hope Tice, Sam Pitluck, Paul Richardson, David Bruce, Lynne Goodwin, Cliff Han, Roxanne Tapia, Elizabeth Saunders, Jeremy Schmutz, Thomas Brettin, Frank Larimer, Miriam Land, Loren Hauser, Stefan Spring, Manfred Rohde, Nikos C. Kyrpides, Natalia Ivanova, Markus Göker, Harry R. Beller, Hans-Peter Klenk, Tanja Woyke

**Affiliations:** 1 DOE Joint Genome Institute, Walnut Creek, California, USA; 2 Los Alamos National Laboratory, Bioscience Division, Los Alamos, New Mexico, USA; 3 Oak Ridge National Laboratory, Oak Ridge, Tennessee, USA; 4 DSMZ – German Collection of Microorganisms and Cell Cultures, Braunschweig, Germany; 5 HZI – Helmholtz Centre for Infection Research, Braunschweig, Germany; 6 Joint BioEnergy Institute (JBEI) and Lawrence Berkeley National Laboratory, Emeryville, California, USA

**Keywords:** facultatively anaerobic, chemoorganotrophic, Gram-negative, non-motile, toluene producer, *Aeromonadaceae*, *Gammaproteobacteria*, JBEI 2008

## Abstract

*Tolumonas auensis* Fischer-Romero *et al.* 1996 is currently the only validly named species of the genus *Tolumonas* in the family *Aeromonadaceae*. The strain is of interest because of its ability to produce toluene from phenylalanine and other phenyl precursors, as well as phenol from tyrosine. This is of interest because toluene is normally considered to be a tracer of anthropogenic pollution in lakes, but *T. auensis* represents a biogenic source of toluene. Other than *Aeromonas hydrophila* subsp. *hydrophila, T. auensis* strain TA 4^T^ is the only other member in the family *Aeromonadaceae* with a completely sequenced type-strain genome. The 3,471,292 bp chromosome with a total of 3,288 protein-coding and 116 RNA genes was sequenced as part of the DOE Joint Genome Institute Program JBEI 2008.

## Introduction

Strain TA 4^T^ (= DSM 9187) is the type strain of the species *Tolumonas auensis* [1], which is the type species of the monotypic genus *Tolumonas* [1,2]. ‘*Tolumonas osonensis*’, isolated from anoxic fresh sediment, was recently proposed as the second species of the genus [3]. ‘*T. osonensis’* does not produce toluene from phenylalanine or other aromatic substrates [3]. The genus name is derived from the Neo-Latin words *toluolum*, toluene, and *monas*, unit, meaning toluene-producing unit. The species epithet originated from the Latin *auensis*, of Lake Au. Strain TA 4^T^ was originally isolated from anoxic sediments of Lake Au (a separate part of Lake Zurich), Switzerland [1]. Four more strains (TA 1-3 and TA5) were also isolated from this source, but these strains were not able to produce toluene [1]. Here we present a summary classification and a set of features for *T. auensis* TA 4^T^, together with the description of the complete genomic sequencing and annotation.

## Classification and features

A representative genomic 16S rRNA sequence of *T. auensis* TA 4^T^ was compared using NCBI BLAST [4] under default settings (e.g., considering only the high-scoring segment pairs (HSPs) from the best 250 hits) with the most recent release of the Greengenes database [5] and the relative frequencies of taxa and keywords (reduced to their stem [6]) were determined, weighted by BLAST scores. The most frequently occurring genera were *Yersinia* (72.3%), *Escherichia* (8.0%), *Tolumonas* (7.2%), *Cronobacter* (6.3%) and *Enterobacter* (3.6%) (219 hits in total). Regarding the ten hits to sequences from members of the species, the average identity within HSPs was 99.3%, whereas the average coverage by HSPs was 98.5%. Among all other species, the one yielding the highest score was *Cronobacter sakazakii* (NC_009778), which corresponded to an identity of 91.8% and an HSP coverage of 100.0%. (Note that the Greengenes database uses the INSDC (= EMBL/NCBI/DDBJ) annotation, which is not an authoritative source for nomenclature or classification.) The highest-scoring environmental sequence was GQ479961 ('changes during treated process sewage wastewater treatment plant clone BXHA2'), which showed an identity of 99.2% and an HSP coverage of 97.9%. The most frequently occurring keywords within the labels of environmental samples which yielded hits were 'reduc' (7.7%), 'sludg' (5.6%), 'activ' (4.8%), 'treatment, wastewat' (4.2%) and 'comamonadacea' (4.1%) (31 hits in total). The most frequently occurring keywords within the labels of environmental samples which yielded hits of a higher score than the highest scoring species were 'reduc' (7.9%), 'sludg' (5.3%), 'activ' (5.0%), 'treatment, wastewat' (4.3%) and 'comamonadacea' (4.3%) (27 hits in total). These keywords fit reasonably well to the ecological properties reported for strain TA 4^T^ in the original description [1].

**Figure 1:**
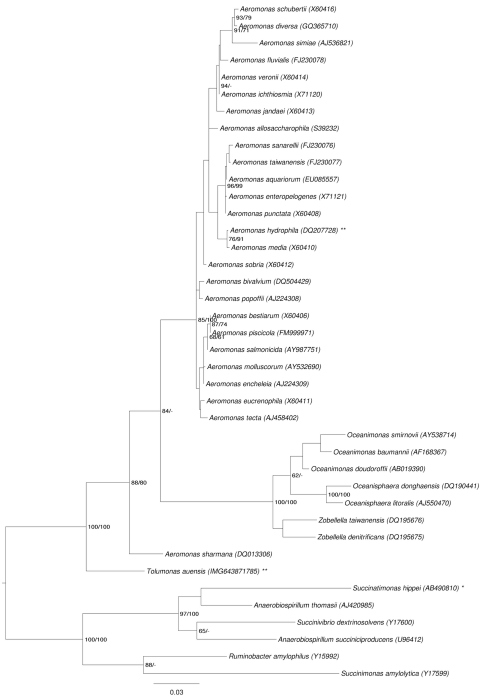
Phylogenetic tree highlighting the position of *T. auensis* relative to the type strains of the other species within the family *Aeromonadaceae*. The tree was inferred from 1,462 aligned characters [7,8] of the 16S rRNA gene sequence under the maximum likelihood (ML) criterion [9] and rooted with the neighboring family *Succinivibrionaceae*. The branches are scaled in terms of the expected number of substitutions per site. Numbers adjacent to the branches are support values from 1,000 ML bootstrap replicates [10] (left) and from 1,000 maximum parsimony bootstrap replicates [11] (right) if larger than 60%. Lineages with type strain genome sequencing projects registered in GOLD [12] are labeled with one asterisk, those also listed as 'Complete and Published' with two asterisks [13].

Figure 1 shows the phylogenetic neighborhood of *T. auensis* in a 16S rRNA-based tree. The sequences of the eight 16S rRNA gene copies in the genome differ from each other by up to 29 nucleotides, and differ by up to 19 nucleotides from the previously published 16S rRNA sequence (X92889), which contains eight ambiguous base calls.

**Figure 2:**
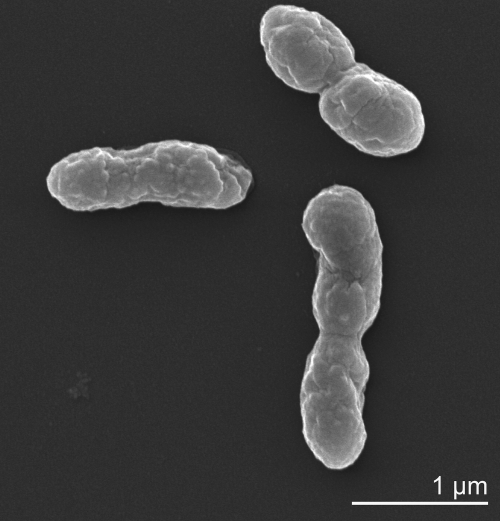
Scanning Electron micrograph of *T. auensis* TA 4^T^

Cells of *T. auensis* strain TA 4^T^ are rod-shaped, 0.9–1.2 × 2.5–3.2 µm (Figure 2, Table1) and occur singly and in pairs [1]. TA 4^T^ cells stain Gram-negative, are non-motile, and grow equally well under oxic and anoxic conditions [1]. Strain TA 4^T^ grows at a pH range from 6.0 to ­7.5, and a temperature range of 12–25°C, with an optimum at 22°C [1]. Oxidase was not produced under any of the growth conditions, whereas catalase was produced only under aerobic conditions [1]. Substrate spectrum and biochemistry of the strain were reported in detail by Fischer-Romero *et al*. [1]. Toluene production was observed under oxic and anoxic conditions, but only in the presence of phenylalanine, phenyllactate, phenylpyruvate, or phenylacetate and one of the carbon sources specified in [1]. Phenol was produced from tyrosine [1].

**Table 1:** Classification and general features of *T.  auensis* according to the MIGS recommendations [14] and the NamesforLife database [15].

**MIGS ID**	**Property**	**Term**	**Evidence code**
	Current classification	Domain *Bacteria*	TAS [16]
		Phylum *Proteobacteria*	TAS [17]
		Class *Gammaproteobacteria*	TAS [18,19]
		Order *Aeromonadales*	TAS [19,20]
		Family *Aeromonadaceae*	TAS [21]
		Genus *Tolumonas*	TAS [1]
		Species *Tolumonas auensis*	TAS [1]
		Type strain TA 4	TAS [1]
	Gram stain	negative	TAS [1]
	Cell shape	rod-shaped	TAS [1]
	Motility	non-motile	TAS [1]
	Sporulation	none	TAS [1]
	Temperature range	mesophile, 12–25°C	TAS [1]
	Optimum temperature	22°C	TAS [1]
	Salinity	not reported	TAS [1]
MIGS-22	Oxygen requirement	facultative	TAS [1]
	Carbon source	various organic acids, sugars and amino acids	TAS [1]
	Energy metabolism	chemoorganotroph	NAS
MIGS-6	Habitat	fresh water	TAS [1]
MIGS-15	Biotic relationship	free living	TAS [1]
MIGS-14	Pathogenicity	none	NAS
	Biosafety level	1	TAS [22]
	Isolation	sediment of a freshwater lake	TAS [1]
MIGS-4	Geographic location	Lake Au, part of Lake Zürich, Switzerland	TAS [1]
MIGS-5	Sample collection time	1993 or before	NAS
MIGS-4.1 MIGS-4.2	Latitude Longitude	47.23 8.63	NAS
MIGS-4.3	Depth	not reported	
MIGS-4.4	Altitude	about 406 m	NAS

Evidence codes - TAS: Traceable Author Statement (i.e., a direct report exists in the literature); NAS: Non-traceable Author Statement (i.e., not directly observed for the living, isolated sample, but based on a generally accepted property for the species, or anecdotal evidence). These evidence codes are from of the Gene Ontology project [23].

### Chemotaxonomy

Data on the cell wall structure of strain TA 4^T^ are not available. Ubiquinones and menaquinones were present under oxic and anoxic conditions, with Q-8 being the major ubiqinone and MK-8 being the major menaquinone [1]. Under aerobic conditions a second, as yet uncharacterized menaquinone was observed [1]. Phosphatidylglycerol and phosphatidyl-ethanolamine were the major phospholipids under both oxic and anoxic growth conditions [1]. The major cellular fatty acids were C_12:0_, C_14:0_, C_16:0_, C_16:1 ω7cis_, C_18:0_, C_18:1 ω7cis_, as well as C_14:0 3-OH_. One half of the latter fatty acid was amide-bound, the other half was ester-linked as were all the other fatty acids [1].

## Genome sequencing and annotation

### Genome project history

This organism was selected for sequencing on the basis of the DOE Joint Genome Institute Program JBEI 2008. The genome project is deposited in the Genomes OnLine Database [12] and the complete genome sequence is deposited in GenBank. Sequencing, finishing, and annotation were performed by the DOE Joint Genome Institute (JGI). A summary of the project information is shown in Table 2.

**Table 2:** Genome sequencing project information

**MIGS ID**	**Property**	**Term**
MIGS-31	Finishing quality	Finished
MIGS-28	Libraries used	Two genomic libraries: Sanger 8 kb pMCL200 and 454 standard libraries
MIGS-29	Sequencing platforms	ABI 3730, 454 GS FLX
MIGS-31.2	Sequencing coverage	5.2 × Sanger, 24.1 × pyrosequencing
MIGS-30	Assemblers	Newbler version 2.0.0-PreRelease-07/15/2008, phrap
MIGS-32	Gene calling method	Prodigal 1.4, GenePRIMP
	INSDC ID	CP001616
	GenBank Date of Release	May 19, 2009
	GOLD ID	Gc01004
	NCBI project ID	33873
	Database: IMG	643692052
MIGS-13	Source material identifier	DSM 9187
	Project relevance	Biotechnology, Biofuel production

### Strain history

The history of strain TA 4^T^ begins with C. Fischer who directly deposited the strain in the DSMZ open collection, where cultures of the strain have been maintained in lyophilized form frozen in liquid nitrogen since 1994.

### Growth conditions and DNA isolation

The culture of strain TA 4^T^, DSM 9187, used to prepare genomic DNA (gDNA) for sequencing was only three transfers removed from the original deposit. A lyophilized sample was cultivated under anoxic conditions at 20°C using DSMZ medium 500 (with 2 g/L glucose as the primary carbon source) [24]. Genomic DNA was isolated using the MasterPure Gram Positive DNA Purification Kit (EpiCentre MGP04100) according to the manufacturer’s instructions. The purity, quality, and size of the bulk gDNA were assessed according to DOE-JGI guidelines. The gDNA ranged in size from 20–125 kb, with most falling in the 75–100 kb range, as determined by pulsed-field gel electrophoresis.

### Genome sequencing and assembly

The genome was sequenced using a combination of Sanger and 454 sequencing platforms. All general aspects of library construction and sequencing can be found at the JGI website [25]. Pyrosequencing reads were assembled using the Newbler assembler (Roche). Large Newbler contigs were broken into 3,816 overlapping fragments of 1,000 bp and entered into assembly as pseudo-reads. The sequences were assigned quality scores based on Newbler consensus q-scores with modifications to account for overlap redundancy and adjust inflated q-scores. A hybrid 454/Sanger assembly was made using the phrap assembler [26]. Possible mis-assemblies were corrected with Dupfinisher and gaps between contigs were closed by editing in Consed, by custom primer walks from sub-clones or PCR products [27]. A total of 764 Sanger finishing reads and four shatter libraries were needed to close gaps, to resolve repetitive regions, and to raise the quality of the finished sequence. The error rate of the completed genome sequence is less than 1 in 100,000. Together, the combination of the Sanger and 454 sequencing platforms provided 29.3 × coverage of the genome. The final assembly contained 20,349 Sanger reads and 409,035 pyrosequencing reads.

### Genome annotation

Genes were identified using Prodigal [28] as part of the Oak Ridge National Laboratory genome annotation pipeline, followed by a round of manual curation using the JGI GenePRIMP pipeline [29]. The predicted CDSs were translated and used to search the National Center for Biotechnology Information (NCBI) non-redundant database, UniProt, TIGRFam, Pfam, PRIAM, KEGG, COG, and InterPro databases. These data sources were combined to assert a product description for each predicted protein. Non-coding genes and miscellaneous features were predicted using tRNAscan-SE [30], RNAMMer [31], Rfam [32], TMHMM [33], and signalP [34].

## Genome properties

**Figure 3:**
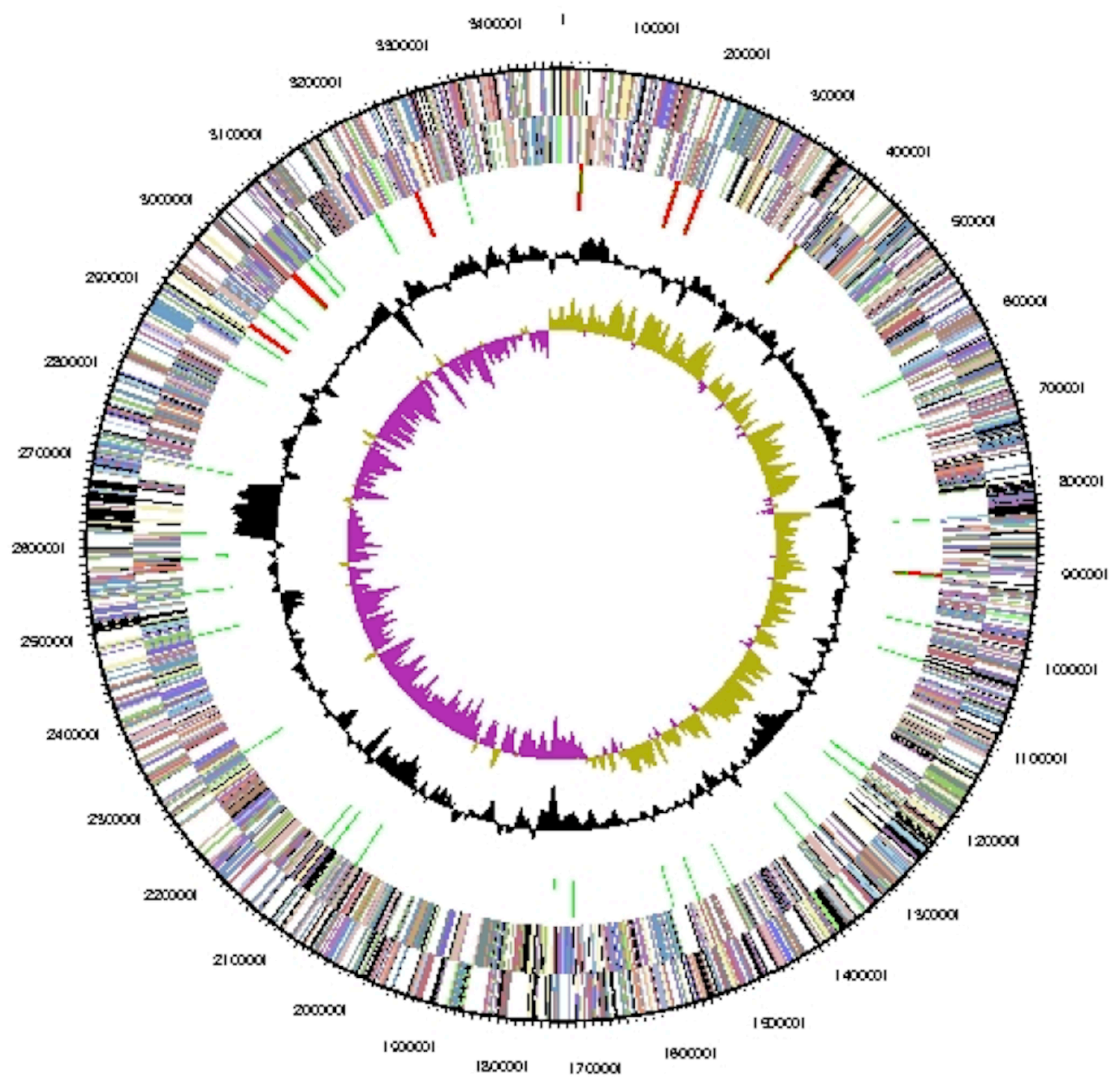
Graphical circular map of the chromosome. From outside to the center: Genes on forward strand (color by COG categories), Genes on reverse strand (color by COG categories), RNA genes (tRNAs green, rRNAs red, other RNAs black), GC content, GC skew.

The genome consists of a 3,471,292-bp long chromosome with a 49.0% G+C content (Table 3 and Figure 3). Of the 3,288 genes predicted, 3,172 were protein-coding genes, and 116 RNAs; 42 pseudogenes were also identified. The majority of the protein-coding genes (76.5%) were assigned a putative function while the remaining ones were annotated as hypothetical proteins. The distribution of genes into COGs functional categories is presented in Table 4.

**Table 3:** Genome Statistics

**Attribute**	**Value**	**% of Total**
Genome size (bp)	3,471,292	100.00%
DNA coding region (bp)	3,122,317	89.95%
DNA G+C content (bp)	1,701,871	49.03%
Number of replicons	1	
Extrachromosomal elements	0	
Total genes	3,288	100.00%
RNA genes	116	3.53%
rRNA operons	8	
Protein-coding genes	3,172	96.47%
Pseudo genes	42	1.28%
Genes with function prediction	2,516	76.52%
Genes in paralog clusters	532	16.18%
Genes assigned to COGs	2,625	79.36%
Genes assigned Pfam domains	2,741	83.76%
Genes with signal peptides	574	17.46%
Genes with transmembrane helices	699	21.26%
CRISPR repeats	1	

**Table 4:** Number of genes associated with the general COG functional categories

**Code**	**value**	**%age**	**Description**
J	171	5.9	Translation, ribosomal structure and biogenesis
A	1	0.0	RNA processing and modification
K	236	8.1	Transcription
L	150	5.2	Replication, recombination and repair
B	0	0.0	Chromatin structure and dynamics
D	36	1.3	Cell cycle control, cell division, chromosome partitioning
Y	0	0.0	Nuclear structure
V	48	1.7	Defense mechanisms
T	124	4.3	Signal transduction mechanisms
M	163	5.6	Cell wall/membrane biogenesis
N	29	1.0	Cell motility
Z	0	0.0	Cytoskeleton
W	0	0.0	Extracellular structures
U	71	2.5	Intracellular trafficking and secretion, and vesicular transport
O	114	3.9	Posttranslational modification, protein turnover, chaperones
C	184	6.4	Energy production and conversion
G	301	10.4	Carbohydrate transport and metabolism
E	241	8.4	Amino acid transport and metabolism
F	70	2.4	Nucleotide transport and metabolism
H	162	5.6	Coenzyme transport and metabolism
I	64	2.2	Lipid transport and metabolism
P	141	4.9	Inorganic ion transport and metabolism
Q	48	1.7	Secondary metabolites biosynthesis, transport and catabolism
R	296	10.2	General function prediction only
S	240	8.3	Function unknown
-	663	20.2	Not in COGs
